# Intraoperative metabolic changes associated with cytoreductive surgery and hyperthermic intraperitoneal chemotherapy

**DOI:** 10.1007/s00423-023-02770-2

**Published:** 2023-01-17

**Authors:** Jesús David Rubio-López, Manuel Durán-Martínez, Andrea Moreno-Blázquez, Lidia Rodríguez-Ortiz, Blanca Rufián-Andújar, Francisca Valenzuela-Molina, Ángela Casado Adam, Juan M. Sánchez-Hidalgo, Sebastián Rufián-Peña, Antonio Romero-Ruiz, J Briceño-Delgado, Álvaro Arjona-Sánchez

**Affiliations:** 1 Department of Anesthesiology, Reina Sofia University Hospital, Cordoba, Spain; 2 Unit of Surgical Oncology, Department of Surgery, Reina Sofia University Hospital, Menendez Pidal Av. 14004, Cordoba, Spain; 3 GE09 Research in Peritoneal and Retroperitoneal Oncological Surgery, Maimonides Biomedical Research Institute of Cordoba (IMIBIC), Reina Sofia University Hospital, University of Cordoba, Cordoba, Spain

**Keywords:** Peritoneal carcinomatosis, HIPEC, Perioperative management, Anesthesia

## Abstract

**Background:**

Cytoreductive surgery (CRS) with hyperthermic intraperitoneal chemotherapy (HIPEC) causes considerable hemodynamic, respiratory, and metabolic changes during the perioperative period.

**Objectives:**

To evaluate metabolic changes associated with this procedure. Understanding perioperative factors and their association with morbidity may improve the perioperative management of patients undergoing this treatment.

**Methods:**

A retrospective review of a prospectively maintained database was performed. All consecutive unselected patients who underwent CRS plus HIPEC between January 2018 and December 2020 (*n* = 219) were included.

**Results:**

The mean age was 58 ± 11.7 years and 167 (76.3%) were female. The most frequent histology diagnosis was serous ovarian carcinoma 49.3% (*n* = 108) and colon carcinoma 36.1% (*n* = 79). Mean peritoneal cancer index was 14.07 ± 10.47. There were significant variations in pH, lactic acid, sodium, potassium, glycemia, bicarbonate, excess bases, and temperature (*p* < 0.05) between the pre-HIPEC and post-HIPEC periods. The closed HIPEC technique resulted in higher levels of temperature than the open technique (*p* < 0.05). Age, potassium level post-HIPEC potassium level, and pre-HIPEC glycemia were identified as prognostic factors for morbidity in multivariate analysis.

**Conclusion:**

The administration of HIPEC after CRS causes significant changes in internal homeostasis. Although the closed technique causes a greater increase in temperature, it is not related to higher morbidity rates. The patient’s age, post-HIPEC potassium level, and pre-HIPEC glycemia are predictive factors for morbidity.

## Introduction

Peritoneal carcinomatosis (PC) is an advanced manifestation of several malignancies related to unfavorable prognosis [1]. The introduction of cytoreductive surgery (CRS) plus the administration of hyperthermic intraperitoneal chemotherapy (HIPEC) has improved the prognosis of these patients [2–5].

CRS-HIPEC has proven to be an effective therapy for malignant tumors to enhance drug delivery in the peritoneal surface after CRS, which may help to eliminate microscopic peritoneal disease [6–13]. The local administration of chemotherapy increases the concentration of cytotoxic agents without systemic toxicity associated [14].

CRS consists of a major intervention that combines peritonectomy procedures and multi-visceral resections to remove all macroscopic tumors from the peritoneal cavity. It is a complex procedure associated with similar morbidity rates to types of major surgeries [15, 16]. During the CRS, the patients develop major changes in the cardiovascular and respiratory systems, requiring specialized anesthetic and surgical management [17]. The hydroelectrolytic and metabolic alterations associated with CRS-HIPEC have been previously reported; however, these studies had a small sample size and did not clarify the effect of these homeostatic modifications on the perioperative outcomes [18–22].

This study aimed to analyze the intraoperative metabolic changes associated with HIPEC administration and their impact on postoperative morbidity in those patients treated with CRS-HIPEC.

## Methods

A retrospective review of a prospectively maintained database was performed. All consecutive unselected patients who underwent CRS plus HIPEC between January 2018 and December 2020 (*n* = 219) were included. The study protocol was approved by Reina Sofia University Hospital Committee for Ethics and Research Cod: 4983 of February 2021.

### Selection of patients for the CRS-HIPEC procedure

All patients included were treated according to the institutionally approved multidisciplinary protocol. Sign-informed consent was obtained. The indications of CRS-HIPEC were as follows: peritoneal carcinomatosis of primary and recurrent ovarian origin FIGO IIIc or IV underwent upfront or interval surgery (when downstaging was confirmed after neoadjuvant treatment), colorectal origin, pseudomyxoma peritonei (PMP), and malignant mesothelioma. Infrequent indications such as carcinomatosis from the endometrial origin, sarcomatosis, gastric cancer, or GISTosis were previously evaluated by the multidisciplinary tumors committee.

The patients included had a good functional status (ECOG) of 0–2. Relative contraindications were age > 70 and body mass index (BMI) > 35 kg/m^2^. Patients considered suitable for CRS + HIPEC who presented relative contraindications were evaluated individually by a multidisciplinary board.

### Surgical procedure

The extent of peritoneal disease was classified using the peritoneal cancer index (PCI) [23]. The principles of cytoreductive surgery and HIPEC have been previously described by Sugarbaker PH [6, 7]. After cytoreduction, HIPEC was administered using two alternative techniques: open (coliseum), which consists of suspending the entire abdominal wall and maintaining a cavity as deep as possible for the administration of HIPEC, or closed technique, which consists of filling the closed abdominal cavity with control to indicate the intra-abdominal volume and is complemented with an intra-abdominal CO_2_ perfusion system to improve the distribution of the HIPEC agent [24]. HIPEC was administered at 41–43 °C temperature. The chemotherapy agents employed were mitomycin C (30 mg/m^2^) for carcinomatosis of intestinal and appendicular origin; paclitaxel (120 mg/m^2^) for gynecological origin; and cisplatin plus doxorubicin (100 mg + 30 mg/m^2^) for peritoneal mesothelioma, sarcoma, and gastric origins. The intraperitoneal solution used for HIPEC was a 1.5% dextrose dilution (4000 mL). The flow rate employed was between 500 and 1000 mL/min. The use of ureteral catheters has been relegated since 2016, and it was used for exceptional cases such as parametria infiltration, previous complex pelvic surgery, or preoperative hydronephrosis [22].

### Intraoperative anesthetic management

Patients were monitored in a standardized protocol for major surgery through ECG (electrocardiogram), NIBP (non-invasive blood pressure), and pulse oximetry before induction. Whenever there was no contraindication, an epidural catheter was placed for a combination of intra and postoperative locoregional anesthesia. Anesthetic depth was monitored using Sendline (Massimo ®). Core body temperature was monitored using an esophageal catheter. Bladder catheterization was performed to quantify urinary output. Finally, arterial and central venous lines were catheterized with ultrasound assistance. During the intervention, a minimally invasive hemodynamic monitor (MostCare Up ®, Vigileo-Flotrac®) was used with a focus on HF (Cardiac Index), PVC (central venous pressure), and PPV (pulse pressure variation) and SVV (variation stroke volume) for upcoming goal-guided fluid delivery [25].

### Variables

Demographic characteristics included the following: patient’s age (years), sex, previous tobacco and alcohol consumption, diabetes mellitus, hypertension, body mass index (BMI), renal function, heart disease, and ASA score. Data tumor-related were the origin of the primary tumor and neoadjuvant chemotherapy received. Intraoperative variables included operative time (min), crystalloids and colloids administered (mL), blood products and vasoactive amine administration, diuresis, chemotherapy agent used for HIPEC, type of HIPEC technique, HIPEC (closed/open), and pre- and post-HIPEC core body temperature (°C). Intraoperative diuresis was classified as (i) < 100 mL during intervention despite diuretic administration, (ii) diuresis forced by diuretics, and (iii) spontaneous diuresis.

Surgical procedure data included PCI, multi-visceral or intestinal resections, and CC score (Sugarbaker’s completeness of cytoreduction score) [26]. Optimal cytoreduction was defined as a CC score of 0–1 since the HIPEC tissue penetration is considered around 2–3 mm. The extension of the peritonectomy procedure was classified according to Sugarbaker’s description: 1–3 regions (partial), 4–6 regions (extended), and more than 6 regions (total) [2].

Pre and post-HIPEC variables: immediately before the start of HIPEC, a blood sample was taken from the patient. At the end of HIPEC, an additional blood sample was obtained to evaluate the homeostatic imbalance caused by HIPEC. The level of pH, lactic acid (mmol/L), excess of base (mmol/L), bicarbonate (mmol/L), sodium (mmol/L), potassium (mmol/L), and glucose (mg/dL) levels were examined on portable blood analyzers (GEM PREMIER 4000 — Werfen ®) in the operating room.

Postoperative outcomes included 30- and 90-day morbidity (Dindo-Clavien classification) [27, 28], hospital length stay (days), and recurrence rate (%). Major morbidity was defined as Dindo-Clavien grade ≥ 3.

### Statistical analysis

Categorical variables were expressed as total numbers and percentages and were compared using a Chi-square or Fisher’s exact test. Continuous data were expressed as means ± standard deviation and were compared using Student’s *t*, Mann Whitney *U*, Welch, or ANOVA tests, according to the distribution of data. Statistical significance was set at *p* < 0.05.

Logistic regression models were performed considering early major complications Dindo-Clavien ≥ 3 (< 30 days) as a dependent variable. All preoperative and intraoperative variables were analyzed as independent variables by univariate analysis, selecting those with *p* < 0.15 for the multivariate analysis. Subsequently, with the selected variables, a multiple logistic regression analysis was performed using the Wald test, and the variables with a *p* < 0.15 (methodical backward selection procedure) were included in the larger model and one by one eliminated from the model. The variables with significance greater than 0.05 were reviewed as possible confounding factors considering them as such if the percentage of change in the coefficients was greater than 15%. As a logistic test for extreme cases, Cook’s distance was used. The Hosmer–Lemeshow statistic based on percentiles was used to assess goodness of fit. SPSS 22.0 (SPSS, Chicago, IL®).

## Results

Between January 2018 and December 2020, a total of 219 patients were undergone CRS + HIPEC. The preoperative demographic parameters are described in Table 1. The mean age was 58 ± 11.7 years and 167 (76.3%) were female. One hundred thirty-eight (62.9%) patients had overweight-obesity. Tumor histologic origins were serous ovarian carcinoma 49.3% (*n* = 108), colorectal 36.1% (*n* = 79), peritoneal pseudomyxoma 7.8% (*n* = 17), endometrium 2.7% (*n* = 6), peritoneal mesothelioma 2.3% (*n* = 4), gastric adenocarcinoma 1.4% (*n* = 3), and others 1.4% (*n* = 2). One hundred fifty-six (71.2%) patients received neoadjuvant chemotherapy with a mean of 2.7 ± 3.19 neoadjuvant cycles received.

**Table 1:** Demographic and preoperative data

Age (years)	58.6 ± 11.7
Gender (Female)	167 (76.3%)
Toxic habits (Tobacco/alcohol)	30 (13.8%) / 9 (4.1%)
Comorbidities	
• Diabetes • AHT • CKD • Cardiopathy • Preoperative creatinine (mg/dL)	23 (10.5%) 58 (26.5%) – 12 (5.5%) 0.7 ± 0.16 mg/dL
ASA	
• I • II • III • IV	31 (14.2%) 97 (44.3%) 85 (38.8%) 6 (2.7%)
BMI	
• Normal weight (18.5–24.9) • Overweight (25–29.9) • Obesity grade I (30–34.9) • Obesity grade II (35–39.9) • Obesity grade III (> 40) • Under weight (< 18.5)	80 (36.5%) 80 (36.5%) 43 (19.6%) 13 (5.9%) 2 (0.9%) 1 (0.5%)
Histology	
• Ovarian -Neoadjuvant chemotherapy • Colorectal -Neoadjuvant chemotherapy • Pseudomyxoma -Neoadjuvant chemotherapy • Endometrium -Neoadjuvant chemotherapy • Peritoneal mesothelioma -Neoadjuvant chemotherapy • Gastric adenocarcinoma -Neoadjuvant chemotherapy • Primary peritoneal -Neoadjuvant chemotherapy	108 (49.3%) 60 (55.6%) 79 (36.1%) 75 (94.9%) 17 (7.8%) 13(76.5%) 6 (2.7%) 2 (33.3%) 4 (1.8%) 2 (50%) 3 (1.4%) 3 (100%) 2 (0.9%) 1 (50%)

*CKD* chronic kidney disease, *AHT* arterial hypertension, *ASA* The American Society of Anesthesiologists, *BMI* body mass index

The intraoperative and postoperative parameters (Tables 2 and 3) were (i) operative time of 383.4 ± 113.3 min, (ii) 81/219 patients had a PCI higher than 20, (iii) 86/219 required a complete peritonectomy, (iv) 134 (61.2%) patients required intestinal resections, (v) optimal cytoreduction was obtained in 213 patients (97.3%), (vi), 36 (16.4%) patients required blood products transfusion during the procedure, (vii) 81 (37%) vasoactive drugs support, and (viii) a total of 175/219 patients had preserved diuresis during the intervention.

**Table 2:** Intraoperative and postoperative data

Operative time (min)	383.4 ± 113.3
PCI score	14.1 ± 10.5
PCI > 20	81 (37%)
Peritonectomy procedures	
• Total • Extended • Partial	86 (39.2%) 73 (33.3%) 60 (27.4%)
Resection of 1 or more intra-abdominal organs	147 (67.1%)
Intestinal resection	134 (61.2%)
CC score	
• CC–0 • CC–1 • CC–2 • CC–3	96 (89.5%) 17 (7.8%) 6 (2.7%) 0
HIPEC technique (open vs closed)	163 (74.4%)/56 (25.6%)
HIPEC time (minutes)	58.8 ± 5.4
HIPEC agents	
• Paclitaxel • Mitomycin–C • Doxorubicin + Cisplatin	116 (52.9%) 96 (43.8%) 7 (3.2%)
Dextrose 1,5% (mL)	2110.4 ± 1693.5
HIPEC flow (mL/min)	929.3 ± 245.4
Major morbidity	
• 30-day • 90-day	49 (22%) 68 (31%)
Mortality	
• 30-day • 90-day	3 (1.4%) 5 (2.3%)
Hospital length of stay (days)	12.1 ± 8.8

*PCI* peritoneal cancer index, *CC score* completeness of cytoreduction score, *HIPEC* hyperthermic Intraperitoneal Chemotherapy

**Table 3:** Intraoperative anesthetic data

Fluid therapy	
• Crystalloids • Colloids	3284.5 ± 1363.7 mL 264.5 mL ± 428.7 mL
Blood products	36 (16.4%)
Vasoactive drugs	81 (37%)
Diuresis:	
• Diuresis < 100 mL • Preserved • Forced with diuretics	5 (2.3%) 175 (79.9%) 39 (17.8%)

According to the Dindo-Clavien morbidity classification, 49/219 (22%) had major complications (grade 3–grade 5) during the first 30 days after surgery, and 68/219 (31%) had cumulative major morbidity within 90 days after surgery. The mean hospital stay was 12.1 ± 8.81 days.

**Fig. 1:**
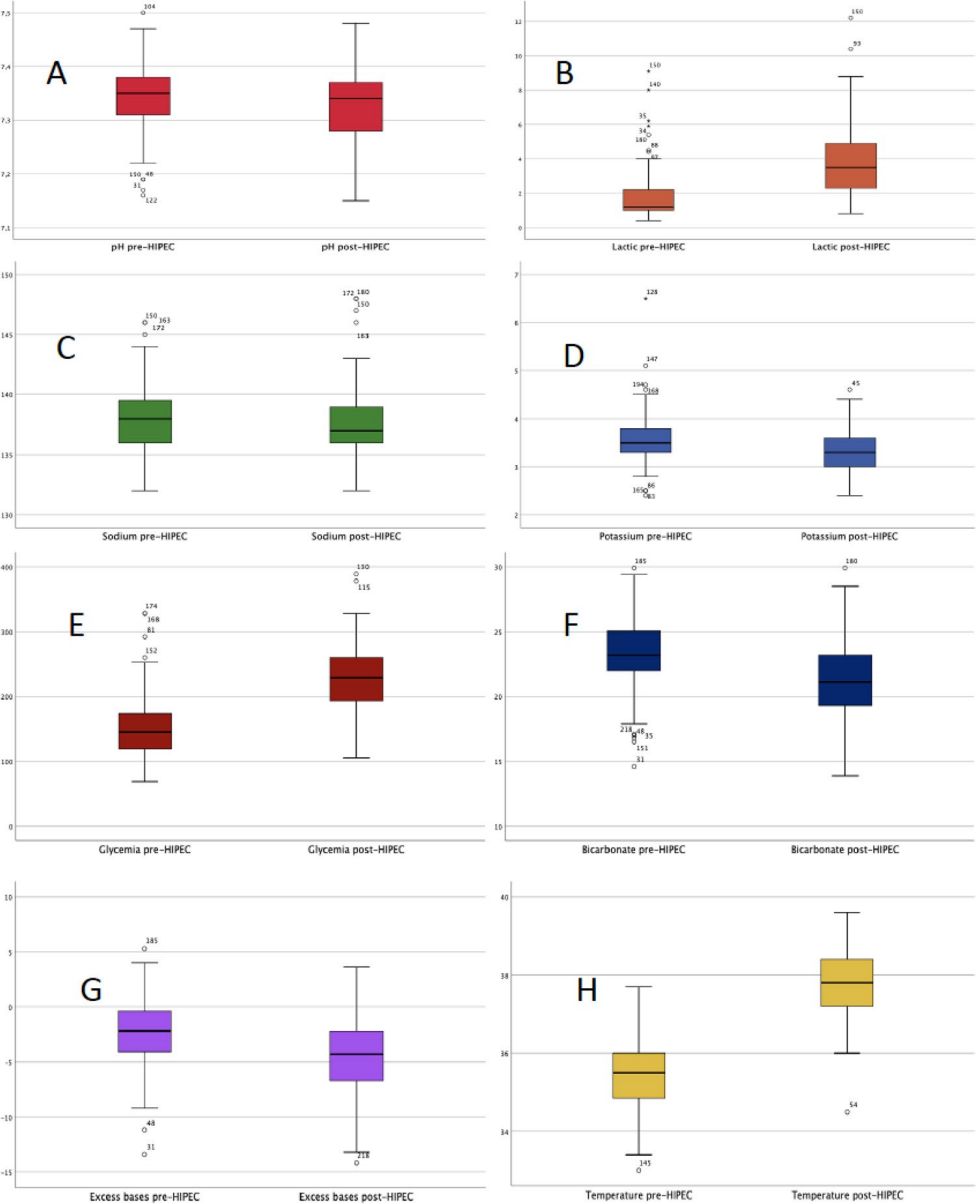
Comparative graph of means and standard deviation of metabolic parameters and temperature before HIPEC versus after HIPEC. **A** pH pre-HIPEC (7.34 ± 0.06) and post-HIPEC (7.32 ± 0.079 (*p* < 0.05). **B** Lactic acid (mmol/L) pre-HIPEC (1.68 ± 1.24) and post-HIPEC (3.72 ± 1.94) (*p* < 0.05). **C** Sodium (mEq/L) pre-HIPEC (138.08 ± 2.72) and post-HIPEC (137.68 ± 3.02) (*p* < 0.05). **D** Potassium (mEq/L) pre-HIPEC (3.57 ± 0.5) and post-HIPEC (3.28 ± 0.42) (*p* < 0.05). **E** Glycemia (mg/dL) pre-HIPEC (149.4 ± 44.79) and post-HIPEC (227.5 ± 50.16) (*p* < 0.05). **F** Bicarbonate (mmol/L) pre-HIPEC (23.25 ± 2.75) and post-HIPEC (21.3 ± 2.94) (*p* < 0.05). **G** Bases excess (mEq/L) pre-HIPEC (− 2.42 ±  − 3.10) and post-HIPEC (− 4.49 ± 3.49) (*p* < 0.05). **H** Temperature (°C) pre-HIPEC (35.47 ± 0.83) and post-HIPEC (37.80 ± 0.79) (*p* < 0.05)

The metabolic changes between the pre- and post-HIPEC included significant variations in blood levels of pH, lactic acid, sodium, potassium, glycemia, bicarbonate, and base excess (*p* < 0.05) (Table 4) (Fig. 1).

**Table 4:** Comparative analysis of metabolic parameters and temperature before HIPEC versus after HIPEC

Parameters	Previous-HIPEC	Posterior-HIPEC	*p*
pH	7.34 ± 0.06	7.32 ± -0.07	0.001
Lactic (mmol/L)	1.68 ± 1.24	3.72 ± 1.94	0.000
Sodium (mEq/L)	138.08 ± 2.72	137.68 ± 3.02	0.007
Potassium (mEq/L)	3.57 ± 0.50	3.28 ± 0.42	0.000
Glycemia (mg/dL)	149.4 ± 44.79	227.5 ± 50.16	0.000
Bicarbonate (mmol/L)	23.25 ± 2.75	21.3 ± 2.94	0.000
Base excess (mEq/L)	− 2.42 ± 3.10	− 4.49 ± 3.49	0.000
Temperature (°C)	35.47 ± 0.83	37.8 ± -0.79	0.000

*HIPEC* hyperthermic intraperitoneal chemotherapy

The comparison between the open vs. closed HIPEC technique showed an increase in temperature in favor of the closed technique (*p* < 0.05). No other significant metabolic changes were observed.

**Fig. 2:**
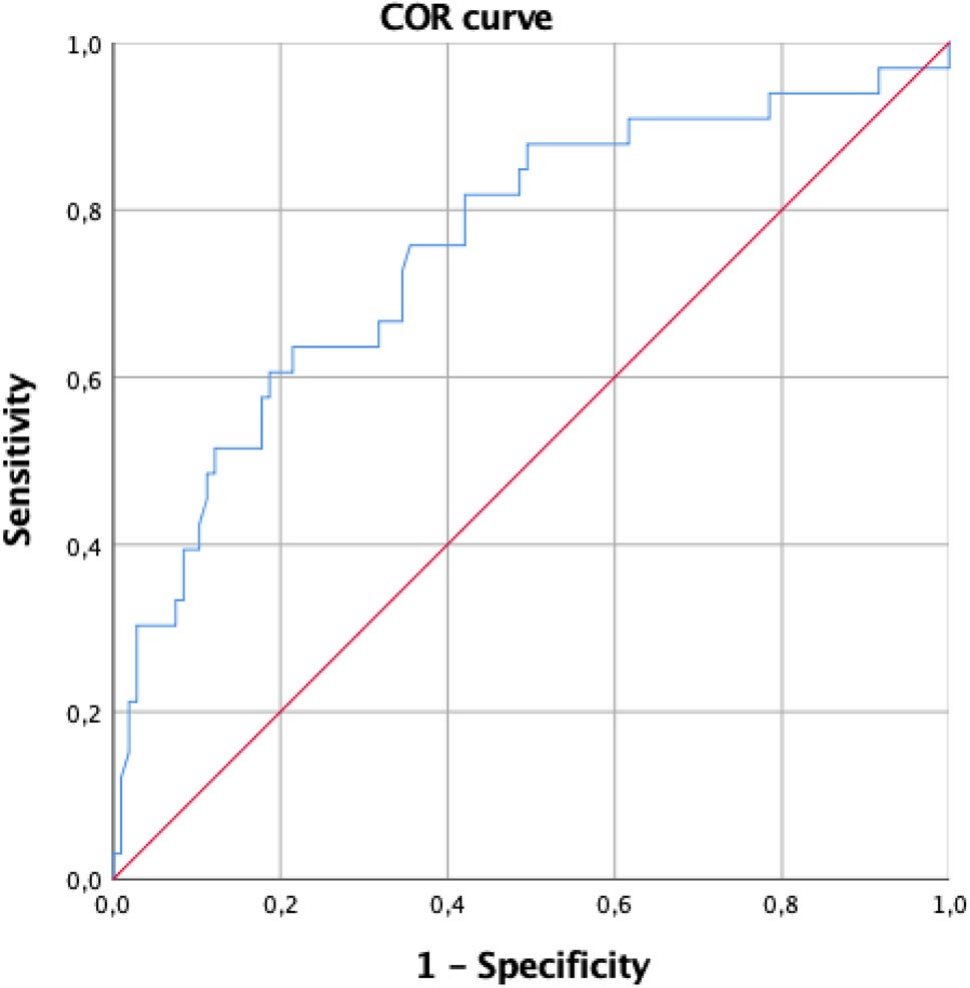
ROC curve for early complications. Area under the ROC curve = 0.718 (95% CI = 0.624 to 0.811). We observed that in almost 72% of all possible pairs of subjects in which one has early major complications and the other does not, the model will assign a higher probability to the subject with early major complications. The resulting logistic equation is *Z* = logit (*p*) =  − 9.780 + age * 0.045 + 1.238 * potassium_posHIPEC + 0.012 * glycemia_preHIPEC

In the multivariate study, patient age, pre-HIPEC glycemia, and post-HIPEC potassium were identified as risk factors for major postoperative complications (Table 5). The area under the ROC curve = 0.718 (95% CI = 0.624 to 0.811) (Fig. 2) showing a higher probability to predict early major complications. The resulting logistic equation is *Z* = logit (*p*) =  − 9.780 + age * 0.045 + 1.238 * potassium_posHIPEC + 0.012 * glycemia_preHIPEC.

**Table 5:** Risk factors associated with early major complications associated with CRS + HIPEC

Variable	Coefficient	Standard error	OR	IC 95%	*P*
Age	0.045	0.019	1.046	1.046–1.086	0.016
Potassium post-HIPEC	1.238	0.471	3.447	1.369–8.678	0.009
Glycemia pre-HIPEC	0.012	0.004	1.012	1.004–1.020	0.005
Constant	− 9.780	2.380	0.000		0.000

## Discussion

In recent years, the CRS + HIPEC has become a routine procedure performed on patients with peritoneal carcinomatosis. This procedure is considered a major surgery since it includes an aggressive surgical technique with HIPEC. HIPEC itself causes artificial ascites and an increase in body temperature causing important changes in internal homeostasis. As a prolonged surgical procedure, the patient experiences considerable blood and fluid losses, requiring specific management during the perioperative period [29–31]. In this sense, there is a lack of information about anesthetic management for these patients. Our study has shown the homeostatic changes and their effect on postoperative outcomes during CRS-HIPEC procedures.

Lactic acid is the end product of the energy pathway working without oxygen due to the impossibility of pyruvate formation under anaerobic conditions, and in other situations, the increase of lactic acid is related to metabolism [32]. The increase in lactic acid levels in patients who have undergone CRS + HIPEC may be due to different mechanisms: (i) the stromal cells, feeding the cancer cells with lactate [33, 34]; (ii) the long duration of the intervention; (iii) the state of peripheral hypoperfusion caused by the surgical aggressiveness and the blood loss; and (iv) the high blood glucose levels. The factors related to HIPEC include (i) the denaturation of serum proteins, (ii) the increasing of intra-abdominal pressure and metabolic requirements, (iii) the peripheral vasodilation due to the effect of hyperthermia, and (iv) the toxicity of the cytostatic used [35, 36].

It is also important to highlight the implication of the type of solution used to dilute the chemotherapeutic agents in the homeostatic changes. Several types of dilutions are used in different worldwide protocols including dextrose solutions or balanced electrolyte solutions [37]. In our protocol, chemotherapy is diluted in a 1.5% dextrose solution resulting in a decrease in sodium, potassium, and pH levels after the completion of HIPEC compared to previously reported using 5% glucose solutions [38]. These differences are probably related to less water reabsorption and less peritoneal electrolyte excretion [39]. Regarding the percentage of dextrose solution used, the increase in glycemia is also affected by surgical stress and resistance to insulin [40]. In this sense, the type of solution should be considered according to the patient’s characteristics [41, 42].

The use of HIPEC in a closed or open way is a controversial issue today, and they have not shown differences in terms of survival or morbidity [43]. When comparing both HIPEC techniques, the analysis of the metabolic parameters and temperature showed that the body temperature at the end of the closed technique was higher (*p* < 0.05) than the open HIPEC technique. Although we did not find significant metabolic changes in our study, a sustained increase in temperature may imply significant hemodynamic and metabolic changes, so strict temperature monitoring is recommended [43, 44].

The observed percentages of major postoperative morbidity (22%) are similar to those previously published by specialized centers [15, 45, 46]. Using the regression model, the variables age, post-HIPEC potassium, and pre-HIPEC blood glucose had a predictive value to detect postoperative major morbidity. Recent studies have failed to correlate intraoperative lactic acid levels with major postoperative morbidity [35]. Previous publications have provided different tools for predicting major morbidity, but they have not included any intraoperative metabolic changes [6, 7, 46, 47].

We are aware that our work has limitations that reduce the power of our conclusions. The first of these deals with all the limitations related to a retrospective study despite prospective data collection. However, this study represents the largest cohort studied for metabolic changes associated with HIPEC procedures.

## Conclusion

The administration of HIPEC after cytoreductive intraperitoneal cancer surgery causes significant changes in internal homeostasis, particularly in the levels of lactic acid, blood glucose, and the patient’s temperature. The patient’s age, post-HIPEC potassium, and pre-HIPEC blood glucose levels are intraoperative predictive factors for major morbidity, and they must be controlled.
